# Factors associated with lifestyle practices for preventing cardiovascular disease in adults aware of metabolic syndrome

**DOI:** 10.20945/2359-4292-2026-0061

**Published:** 2026-06-08

**Authors:** Jong Sun Ok, Kyung-Jin Kim, Kyung-Hee Kim, Do Kyeong Song, Kyung Jin Ahn, Chan Seok Park, Mi-Seung Shin

**Affiliations:** 1 Department of Nursing, Konkuk University, Chungju, Korea; 2 Division of Cardiology, Department of Internal Medicine, Ewha Womans University College of Medicine, Seoul, Korea; 3 Division of Cardiology, Incheon Sejong Hospital, Incheon, Korea; 4 Department of Internal Medicine, Ewha Womans University School of Medicine, Seoul, Korea; 5 Department of Pediatrics, Gachon University Gil Medical Center, Incheon, Korea; 6 Division of Cardiology, Department of Internal Medicine, Bucheon St Mary’s Hospital, The Catholic University of Korea, Seoul, Korea; 7 Division of Cardiology, Department of Internal Medicine, Gachon University Gil Medical Center, Incheon, Korea

**Keywords:** Healthy lifestyle, metabolic syndrome, risk factor, adult, abdominal obesity

## Abstract

**Objective:**

The global increase in metabolic syndrome (MetS) highlights the need for effective lifestyle interventions to reduce cardiovascular disease (CVD) risk. This study aimed to identify factors associated with lifestyle behaviors for preventing CVD complications among adults aware of MetS.

**Subjects and methods:**

A cross-sectional online survey was conducted from January to February 2023 among 1,000 South Korean adults aged 20-69 years. After excluding 212 participants unaware of MetS, 788 respondents were included, of whom 710 engaged in at least three of nine recommended lifestyle behaviors: smoking cessation, alcohol abstinence, body weight monitoring, waist circumference measurement, blood pressure monitoring, regular hospital visits, adequate sleep, adherence to a low-salt diet, and regular physical activity. Participants were categorized into those with one or more MetS risk factors (META) and those without (Non-META).

**Results:**

participants with MetS risk factors were predominantly male and aged 50-69 years, while those without were more likely female and aged 20-39 years. Abdominal obesity was the most common risk factor, whereas waist circumference monitoring was the least practiced behavior. The META group showed higher rates of blood pressure monitoring and hospital visits, while the Non-META group reported more frequent smoking cessation and alcohol abstinence.

**Conclusion:**

Engagement in healthy lifestyle behaviors was positively associated with female sex, older age, and higher awareness of MetS-related complications. The limited practice of waist circumference monitoring underscores the need for targeted education and personalized preventive strategies to enhance CVD risk reduction among individual aware of MetS.

## INTRODUCTION

Metabolic syndrome (MetS) has emerging as a major global public health concern, characterized by a cluster of interrelated metabolic abnormalities that substantially increase the risk of chronic diseases (^1^). Epidemiological evidence indicates that MetS affects approximately one-quarter to one-third of the global population, with prevalence rates continuing to rise across diverse regions (^2^). National data report prevalence estimates of 34.2% in the United States, 29.6% in Brazil, and 21% in South Korea, underscoring its widespread burden (^3^-^5^).

Clinically, MetS is defined by the presence of at least three of the following five criteria: abdominal obesity, elevated fasting blood glucose, elevated blood pressure, elevated triglycerides, and reduced high-density lipoprotein (HDL) cholesterol level (^1^). This constellation of metabolic risk factors is strongly associated with an increased incidence of cardiovascular disease (CVD), type 2 diabetes mellitus, cognitive decline, and all-cause mortality (^1^). Moreover, the cumulative presence of multiple MetS components has been shown to exponentially elevate the risk of severe complications, including CVD events, diabetic complications, and stroke (^6^).

The etiology of MetS is multifactorial, arising from complex interactions among genetic predisposition and modifiable behaviors, psychosocial, and environmental factors such as diet, physical inactivity, stress, socioeconomic status (^7^). Among these, lifestyle behaviors represent key modifiable determinants for both the prevention and management of MetS (^8^). Current clinical guidelines, including the National Cholesterol Education Program Adult Treatment Panel (NCEP ATP) III, recommend lifestyle modification-comprising dietary regulation, increased physical activity, and weight control-as the first-line strategy for MetS management (^9^).

However, the interdependence among lifestyle behaviors present challenges in both assessment and intervention. Prior research has largely examined isolated behavioral or clinical components, limiting compressive understanding of how multiple lifestyle factors interact to influence MetS outcomes (^8^). A more integrated approach is therefore essential to elucidate behavioral patterns underlying lifestyle adherence and to develop effective, sustainable preventive strategies (^8^).

Awareness of MetS constitutes a critical precursor to behavioral change, serving as the foundation for adopting preventive health behaviors (^10^). Previous studies have demonstrated that individuals aware of MetS are more likely to engage in lifestyle modification aimed at mitigating associated risk factors (^10^). Enhanced awareness may further facilitate early detection and management of metabolic abnormalities, thereby reducing the risk of CVD―the more critical complication of MetS (^1^). Accordingly, the present study aimed to identify factors associated with lifestyle practices for the prevention of CVD among adults who are aware of MetS.

## SUBJECTS AND METHODS

### Study design and data collection

This study employed a cross-sectional design and was conducted from January to February 2023. With the exception of study design development, all survey administration procedures were implemented by MegaResearch, a professional research agency based in Seoul, South Korea. As of December 2022, the estimated adult population aged ≥20 years in South Korea was 37,121,412. To obtain a nationally representative sample of adults aged 20-69 years, a total of 1,000 participants were recruited using a stratified systematic sampling method based on sex (male/female), age group (20-29, 30-39, 40-49, 50-59, and 60-69 years), and geographical region (eight metropolitan and eight non-metropolitan areas).

Data were collected via structured, self-administered online questionnaires. Survey invitations containing a unique link to the web-based instrument were distributed to randomly selected panel members registered with the research agency. Participants completed the questionnaire independently, and responses were automatically recorded on a secure data server. To minimize item non-response, the survey system required participants to complete each question before proceeding to the next. All aspects of data collection were monitored in real time by the research agency to ensure data integrity, prevent duplicate entries, and maintain response accuracy (^11^).

### Study population

The study population was derived from the nationally representative sample of South Korea adults through stratified sampling by sex, age, and region of residence to ensure demographic representativeness. Eligible participants were adults aged 20 to 69 years who provided informed consent and completed the online questionnaire after confirming full understanding of the study purpose and procedures. Exclusion criteria were as follows: (^1^) individuals younger than 20 or older than 69 years, (^2^) individuals without adequate internet access, (^3^) those who declined participation, (^4^) respondents who failed to complete the final survey item; and (^5^) participants from specific sex-age strata exceeding the predetermined sample quota (^11^).

Participants who reported being unaware of MetS (n = 212) were excluded, resulting in a final analytical sample of 788 individuals with MetS awareness. Of these, 710 participants who reported engaging in at least three of the following nine lifestyle behaviors were included in the final analysis: smoking cessation, alcohol abstinence, body weight monitoring, waist circumference measurement, blood pressure monitoring, regular physical activity or adherence to a low-salt diet-recognized. Smoking cessation was considered essential lifestyle behavior for preventing CVD complications. Additionally, participants were required to engage in at least one of the two key lifestyle modifications―regular physical activity or adherence to a low-salt diet―recognized as critical for preventing MetS-related complications. In addition to these core behaviors, participants were required to practice at least one additional lifestyle behavior from the remaining items.

Participants were classified into two groups: those with one or more MetS risk factors (hypertension, hyperlipidemia, diabetes mellitus, or abdominal obesity) (META) and those without any risk factors (Non-META).

### Questionnaire

Demographic variables included age, sex, educational attainment, monthly household income, occupation, marital status, presence of comorbidities, and family history of CVD. Comorbid conditions-specifically hypertension, diabetes mellitus, hyperlipidemia, and abdominal obesity-were evaluated as indicator of MetS risk factors.

MetS-related variables comprised the following: awareness of MetS (1 = “Very well aware”, 2 = “Well aware”, 3 = “Somewhat aware”, 4 = “Unaware”, 5 = “Completely unaware”); awareness of the association between MetS and CVD complications (1 = “Strongly agree”, 2 = “Somewhat agree”, 3 = “Slightly agree”, 4 = “Do not agree”, 5 = “Do not agree at all”); and frequency of waist circumference and body weight monitoring within the past three years (1 = “Frequently”, 2 = “Occasionally”, 3 = “Rarely”, 4 = “Never”). The questionnaire also assessed engagement in lifestyle modification behaviors for MetS prevention and perceived barriers to implementing such behaviors.

Lifestyle practices for MetS prevention included blood pressure monitoring, regular hospital visits, adequate sleep, adherence to a low-salt diet, regular physical activity, vitamin supplementation, and dietary supplement intake. Participants who reported frequent monitoring of waist circumference and body weight during the previous three years were also classified as engaging in preventive lifestyle practices.

Smoking cessation and alcohol abstinence were assessed as additional lifestyle behaviors related to comorbidity management. Participants who reported abstinence from smoking or alcohol consumption were categorized as practicing these behaviors. Barriers to the adoption of MetS-related lifestyle practices were classified into seven categories: financial constraints, perception of already maintaining a healthy lifestyle, lack of time, excessive workload, insufficient knowledge of appropriate behaviors, disbelief in the effectiveness of lifestyle modification, and other unspecified factors.

Participants with one or more comorbidities (hypertension, diabetes mellitus, hyperlipidemia, or abdominal obesity) were categorized as the MetS risk factor group, whereas those without any of these conditions were classified as the non-risk factor group.

Given the established importance of smoking cessation in the prevention of non-communicable diseases and overall health promotion (^12^), this behavior was included as a core component of CVD-preventive lifestyle practices. Comprehensive lifestyle modification was operationally defined as engagement in at least three lifestyle behaviors (^13^), including one of either low-salt diet adherence or regular physical activity, in addition to at least one other behavior (excluding vitamin or supplement intake). The detailed questionnaire is provided in **Supplementary Material**.

### Ethics

This study was reviewed and approved by the institutional Review Board (IRB) of the Gachon University Medical Center (IRB No. GFIRB2023-084). Given that the survey was conducted anonymously and did not involve the collection of personally identifiable information, the IRB waived the requirement for written informed consent. All participants provided implied consent by voluntarily completing the online questionnaire.

### Statistical analysis

Descriptive statistics were used to summarize the participants’ demographic characteristics and MetS-related variables. Categorical variables were compared using the chi-square test, while continuous variables were assessed with the independent samples t-test. Multivariate logistic regression analysis was performed to identify factors independently associated with engagement in healthy lifestyle behaviors. All statistical tests were two-tailored, and a significance threshold of p < 0.05 was applied. Data analysis were conducted using R software version 4.2.0 for windows (The Comprehensive R Archive Network, Auckland, New Zealand).

## RESULTS

### General characteristics of participants and metabolic syndrome-related variables

The general characteristics of the study population and response to MetS-related questionnaire items are summarized in **Table 1**. The mean age of participants in MetS risk factor group (META) was significantly higher than that of the non-risk factor group (Non-META) (49.41 ± 12.60 years vs. 43.11 ± 13.02 years; t = 6.27, *p* < 0.001). Age distribution differed between groups: the META group had a higher proportion of individuals in their 50s and 60s, whereas the Non-META group had a higher proportion of participants in their 20s and 30s. Sex distribution also differed significantly, with male predominating in the META group (54.0%) and females in the non-META group (55.9%) (x^2^ =39.20, *p* < 0.001). Marital status showed a significant difference, with a higher proportion of married participants in the META group (72.2% ) compared with the Non-META group (51.0%) (x^2^ = 40.90, *p* < 0.001). Educational attainment did not differ significantly, with the majority holding a college degree or higher (x^2^ = 2.66, *p* = 0.264). Similarly, monthly household income distribution was not significantly different between groups, with the largest proportion earning ≥ KRW 5.00 million (x^2^ = 7.66, *p* = 0.176).

**Table 1:** General characteristics of participants and Metabolic syndrome-related variables, n = 710

Variables		META N=463	Non-META N=247	x^2^ or t	p
		n (%) or M±SD	n (%) or M±SD		
Age		49.41±12.60	43.11±13.02	6.27	<0.001^*^
20s		46 (9.9)	56 (22.7)	39.20	<0.001^*^
30s		61 (13.2)	51 (20.6)		
40s		94 (20.3)	52 (21.1)		
50s		132 (28.5)	50 (20.2)		
60s		130 (28.1)	38 (15.4)		
Sex	Male	250 (54.0)	109 (44.1)	5.88	0.015^*^
	Female	213 (46.0)	138 (55.9)		
Marital status	Single	96 (20.7)	107 (43.3)	40.90	<0.001^*^
	Married	339 (72.2)	126 (51.0)		
	Divorce/Bereave/Separate	28 (6.0)	14 (5.7)		
Education	≤Middle school	5 (1.0)	2 (0.8)	2.66	0.264
	High school	110 (23.8)	46 (18.6)		
	≥College	348 (75.2)	199 (80.6)		
Monthly household income (KRW)	≤100	15 (3.3)	7 (2.8)	7.66	0.176
	100-199	32 (6.9)	16 (6.5)		
	200-299	75 (16.2)	53 (21.4)		
	300-399	95 (20.5)	47 (19.0)		
	400-499	89 (19.2)	31 (12.6)		
	≥500	157 (33.9)	93 (37.7)		
Occupation	None	35 (7.5)	19 (7.7)	16.95	0.031^*^
	Self-employment	32 (6.9)	25 (10.1)		
	Sales/Services	42 (9.1)	21 (8.5)		
	Technical	41 (8.9)	25 (10.1)		
	Office work	131 (28.3)	85 (34.4)		
	Administration/Management	39 (8.4)	8 (3.3)		
	Professional/Public official/Teacher	36 (7.8)	24 (9.7)		
	Housewife	79 (17.1)	25 (10.1)		
	Part time/Freelancer/College/Graduate student	28 (6.0)	15 (6.1)		
Family history of cardiovascular disease	Yes	139 (30.0)	25 (10.1)	34.80	<0.001^*^
	No	324 (70.0)	222 (89.9)		
Awareness of the association between MetS and CVD complications	Strongly agree	207 (44.7)	83 (33.6)	9.84	0.043^*^
	Somewhat agree	152 (32.8)	100 (40.5)		
	Slightly agree	75 (16.2)	45 (18.2)		
	Do not agree	2 (0.4)	0 (0.0)		
	Dd not agree at all	27 (5.9)	19 (7.7)		
Types of lifestyle practice (double check)	Waist circumference	38 (8.2)	22 (8.9)	0.031	0.859
	Body weight	192 (41.5)	103 (41.7)	0.000	1.000
	Blood pressure	135 (29.2)	32 (13.0)	22.61	<0.001
	Alcohol abstinence	306 (66.1)	201 (81.4)	17.69	<0.001
	Smoking cessation	325 (70.2)	214 (86.6)	22.93	<0.001
	Physical activity	254 (54.9)	152 (61.5)	2.67	0.102
	Adequate sleep	153 (33.1)	79 (32.0)	0.041	0.839
	Low salt diet	156 (33.7)	100 (40.5)	2.93	0.087
	Routine medical checkup	172 (37.2)	41 (16.6)	31.42	<0.001
	Taking health supplements (vitamins, red ginseng, etc.)	225 (48.6)	98 (39.7)	3.79	0.051
barriers to practicing lifestyle	Maintaining a healthy lifestyle	67 (14.5)	42 (17.0)	5.12	0.401
	Economic burden	95 (20.5)	47 (19.0)		
	Lack of time	53 (11.5)	39 (15.8)		
	Too much to do	66 (14.3)	29 (11.7)		
	Not sure what to do	147 (31.8)	64 (25.9)		
	Lifestyle modification don’t help	27 (5.8)	22 (8.9)		
	Others	8 (1.7)	4 (1.6)		

* p < 0.05, MetS: metabolic syndrome risk factor; CVD: cardiovascular disease; META: those with one or more MetS risk factors (hypertension, hyperlipidemia, diabetes mellitus, or abdominal obesity); Non-META: those without any risk factors

Occupational status differed significantly (x^2^ = 16.95, *p* = 0.031). Office workers constituted the largest occupational category in both groups. The META group included a higher proportion of housewives and sales/service workers, whereas the Non-META group included a higher proportion of self-employed and technical workers

Regarding family history of CVD, a significantly greater proportion of participants in the META group reported a positive history compared with the Non-META group (30.0% vs. 10.1%; x^2^ = 34.80, *p* < 0.001). Awareness of the relationship between MetS and CVD complications was also higher in the META group, with 44.7% reporting strong awareness versus 33.6% in the Non-META group (x^2^ = 9.84, *p* = 0.043).

**Figure 1:**
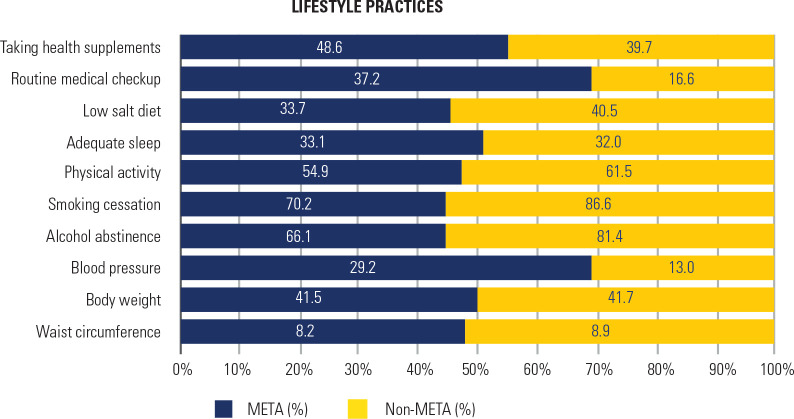
**Comparisons of lifestyle practices between participants with and without metabolic syndrome risk factors.** Participants with metabolic syndrome risk factors (META group) demonstrated significantly higher rates of blood pressure monitoring and regular hospital visits (*p* < 0.001), whereas those without metabolic syndrome risk factors (Non-META group) exhibited higher rates of alcohol abstinence and non-smoking (*p* < 0.001).

In terms of lifestyle practices, participants in the META group reported higher rates of blood pressure monitoring and regular hospital visits (x^2^ = 22.61, *p* < 0.001; x^2^ = 31.42, *p* < 0.001), whereas participants in the Non-META group reported higher rates of alcohol abstinence and smoking cessation (x^2^ = 17.69, *p* < 0.001; x^2^ = 22.93, *p* < 0.001) (**Figure 1**).

**Figure 2:**
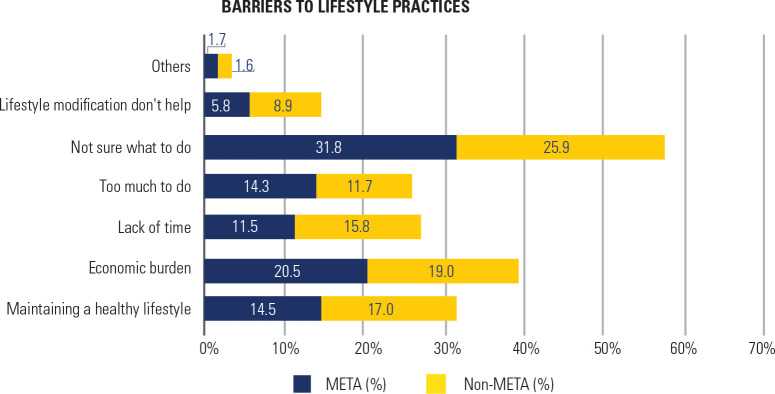
**Comparisons of barriers to lifestyle practices between participants with and without metabolic syndrome risk factors.** The most frequently reported barrier to lifestyle practice in both groups was a lack of knowledge regarding how to implement healthy lifestyle behaviors for the prevention of metabolic syndrome (*p* = 0.401).

Although no statistically significant differences were observed between groups in perceived barriers to lifestyle modification, the most frequently reported barrier in both groups was insufficient knowledge regarding appropriate lifestyle practices (x^2^ = 5.12, *p* = 0.401) (**Figure 2**).

### Comparison of lifestyle practices and barriers according to types of metabolic syndrome risk factors

The MetS risk factors examined in this study included hypertension, hyperlipidemia, diabetes mellitus, and abdominal obesity. Lifestyle practices and perceived barriers to lifestyle modification stratified by these risk factors are summarized in **Table 2**. The prevalence of MetS risk factors among participants was 48.0% for hypertension, 50.3% for hyperlipidemia, 21.8% for diabetes, and 66.5% for abdominal obesity.

**Table 2:** Comparison of Lifestyle practices and barriers according to types of Metabolic syndrome risk factors, n = 710

Variables		MetS risk factors (double check)					
		HTN	Hyperlipidemia	DM	Abdomen obesity	x^2^	p
		n (%)	n (%)	n (%)	n (%)		
		222 (48.0)	233 (50.3)	101 (21.8)	308 (66.5)		
Types of lifestyle practice (double check)	Waist circumference	22 (9.9)	23 (9.9)	13 (12.9)	25 (8.1)	2.63	0.452
	Body weight	94 (42.3)	113 (48.5)	47 (46.5)	127 (41.2)	1.97	0.578
	Blood pressure	96 (43.2)	82 (35.2)	40 (39.6)	84 (27.3)	45.21	<0.001^*^
	Alcohol abstinence	142 (64.0)	152 (65.2)	65 (64.4)	187 (60.7)	1.13	0.769
	Smoking cessation	151 (68.0)	162 (69.5)	63 (62.4)	206 (66.9)	1.46	0.691
	Physical activity	120 (54.1)	141 (60.5)	57 (56.4)	170 (55.2)	1.49	0.685
	Adequate sleep	64 (28.8)	70 (30.0)	36 (35.6)	111 (36.0)	3.45	0.328
	Low salt diet	79 (35.6)	86 (36.9)	36 (35.6)	103 (33.4)	2.67	0.444
	Routine medical checkup	104 (46.9)	102 (43.8)	49 (48.5)	107 (34.7)	25.91	<0.001^*^
	Taking health supplements (vitamins, red ginseng, etc.)	101 (45.5)	126 (54.1)	41 (40.6)	142 (46.1)	15.82	0.001^*^
barriers to practicing lifestyle	Maintaining a healthy lifestyle	38 (17.1)	34 (14.6)	22 (21.8)	36 (11.7)	12.14	0.669
	Economic burden	42 (18.9)	56 (24.0)	22 (21.8)	71 (23.1)		
	Lack of time	24 (10.8)	24 (10.3)	16 (15.8)	38 (12.3)		
	Too much to do	32 (14.4)	26 (11.2)	13 (12.9)	48 (15.6)		
	Not sure what to do	68 (30.6)	77 (33.1)	21 (20.8)	90 (29.2)		
	Lifestyle modification don’t help	15 (6.8)	10 (4.3)	6 (5.9)	18 (5.8)		
	Others	3 (1.4)	6 (2.6)	1 (1.0)	7 (2.3)		

* p < 0.05, MetS: metabolic syndrome.

**Figure 3:**
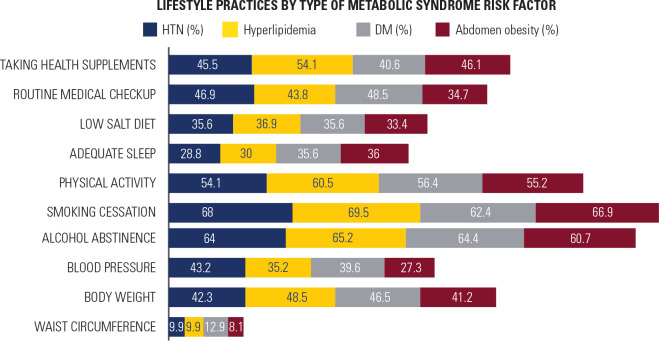
**Comparison of lifestyle practices by type of metabolic syndrome risk factor.** Four metabolic syndrome risk factors were examined: hypertension, hyperlipidemia, diabetes, and abdominal obesity. Among lifestyle practice items, the rate of blood pressure monitoring was the highest among participants with hypertension (*p* < 0.001), and regular hospital visits were the most frequent among those with diabetes (*p* < 0.001). Consumption of health supplements was highest among participants with hyperlipidemia (*p* = 0.001).

Among lifestyle practices, blood pressure monitoring was most frequently reported by participants with hypertension (x^2^ = 45.21, *p* < 0.001), whereas regular hospital visits were most common among those with diabetes (x^2^ = 25.91, *p* < 0.001). Additionally, consumption of health supplements, including vitamins and red ginseng, was highest among individuals with hyperlipidemia (x^2^ = 15.82, *p* = 0.001) (**Figure 3**).

**Figure 4:**
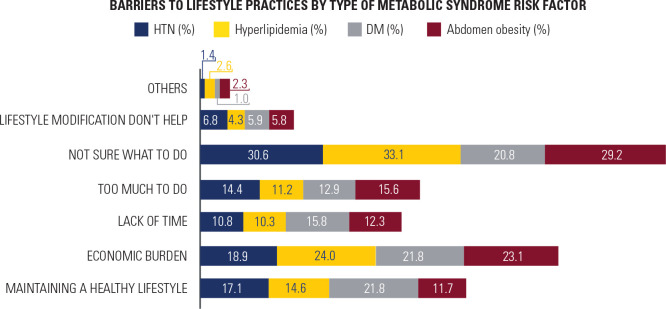
**Comparison of barriers to lifestyle practices by type of metabolic syndrome risk factor.** Four metabolic syndrome risk factors were examined: hypertension, hyperlipidemia, diabetes, and abdominal obesity. Among participants with diabetes, the primary barrier to adopting healthy lifestyle behaviors was financial burden, whereas for the other risk factor groups, the predominant barrier was a lack of knowledge regarding how to implement healthy lifestyle behaviors for the prevention of metabolic syndrome (p = 0.669).

Although no statistically significant differences were observed in reported barriers to lifestyle modification across MetS risk factors (x^2^ = 12.14, *p* = 0.669), the most frequently reported barrier among participants with hypertension, hyperlipidemia, and abdominal obesity was uncertainly regarding how to implement appropriate lifestyle changes. In contrast, financial burden was the predominant barrier among participants with diabetes (**Figure 4**).

### Factors associated with lifestyle practices for the prevention of cardiovascular disease in adults aware of metabolic syndrome

Multivariate logistic regression analysis (**Table 3**) identified several significant predictors of engagement in heathy lifestyle practice. Female sex, older age, and greater awareness of complications associated with MetS were positively and independently associated with adherence to recommended lifestyle behaviors. These findings suggest that demographic factors and knowledge of MetS-related risks play a critical role in promoting preventive health behaviors among adults aware of their metabolic risk status.

**Table 3:** Factors associated with Lifestyle practices for the prevention of Cardiovascular disease in adult aware of Metabolic syndrome, n = 710

Variables	Estimate	SE	Z	P	OR	CI
Age	0.028	0.009	3.161	0.002^*^	1.03	1.01-1.05
Sex (Reference = Male)	0.383	0.187	2.045	0.041^*^	1.47	1.02-2.12
Education (Reference = Middle school)	-0.255	0.202	-1.262	0.207	0.77	0.52-1.15
Monthly household income (Reference = ≤100)	0.076	0.066	1.166	0.244	1.08	0.95-1.23
Occupation_Housewife (Reference = None)	0.228	0.412	0.553	0.581	1.26	0.56-2.82
Occupation_ Part time/Freelancer/students (Reference = None)	-0.488	0.439	-1.112	0.266	0.61	0.26-1.45
Occupation_ Public official/Teacher/Professional (Reference = None)	-0.163	0.419	-0.388	0.698	0.85	0.37-1.93
Occupation_ Administration/Management (Reference = None)	-0.987	0.464	-2.218	0.033	0.37	0.15-0.92
Occupation_ Technical (Reference = None)	-1.551	0.425	-3.652	0.000	0.21	0.09-0.49
Occupation_ Sales/Services (Reference = None)	-0.917	0.412	-2.226	0.026	0.40	0.18-0.90
Occupation_ Office work (Reference = None)	-0.472	0.349	-1.355	0.175	0.62	0.31-1.23
Occupation_ Self-employment (Reference = None)	-0.662	0.416	-1.593	0.111	0.52	0.23-1.16
Family history of cardiovascular disease (Reference = No)	-0.662	0.208	-3.177	0.001	0.52	0.34-0.78
Marital status_Single (Reference = Divorce/Bereave/Separate)	0.458	0.411	1.115	0.265	1.58	0.71-3.54
Marital status_Married (Reference = Divorce/Bereave/Separate)	0.409	0.370	1.106	0.269	1.51	0.73-3.11
Awareness of MetS complications (Reference = Unknown)	0.255	0.094	2.709	0.007^*^	1.29	1.07-1.55
Barriers to practicing lifestyle_ Economic burden (Reference = Maintaining a healthy lifestyle)	-0.221	0.287	-0.768	0.442	0.80	0.46-1.41
Barriers to practicing lifestyle_ Lack of time/Too much to do (Reference = Maintaining a healthy lifestyle)	0.036	0.269	0.134	0.893	1.04	0.61-1.76
Barriers to practicing lifestyle_Not sure what to do (Reference = Maintaining a healthy lifestyle)	-0.304	0.265	-1.148	0.251	0.74	0.44-1.24
Barriers to practicing lifestyle_Lifestyle modification don’t help (Reference = Maintaining a healthy lifestyle)	0.184	0.386	0.477	0.633	1.20	0.56-2.56
Barriers to practicing lifestyle_Other (Reference = Maintaining a healthy lifestyle)	1.227	0.824	1.489	0.136	3.41	0.68-17.15
No. risk factors for metabolic syndrome	-0.340	0.080	-4.238	<.001	0.71	0.61-0.83
Null deviance: 983.99 Residual deviance: 868.12 AIC: 914.12						

* p < 0.05.

## DISCUSSION

This study investigated the prevalence of MetS risk factors, lifestyle practices, determinants of lifestyle adherence among a nationally representative sample of adults in South Korea. Among MetS risk factors, abdominal (central) obesity was the most prevalent in the META group. Central obesity, a core component of MetS, is influenced by hormonal factors such as gonadal steroids (^14^) and individuals of Asian descent are known to exhibit greater visceral adiposity compared to Caucasians at equivalent body mass indices (BMI) (^15^). The global increase in obesity has substantially contributed to the rising burden of MetS (^1^).

Despite the central role of abdominal obesity in MetS pathogenesis, waist circumference monitoring―a key metric for assessing central adiposity―was the least frequently practiced lifestyle behavior in both META and Non-META groups. Primary barriers identified was limited knowledge regarding appropriate lifestyle practices. Practical challenge, including privacy concerns, variability in seasonal clothing, and inconsistencies in clinical measurement techniques, may also hinder regular waist circumference monitoring. These findings highlight the need for targeted educational initiatives directed at both healthcare providers and the public, emphasizing waist circumference assessment alongside standard metrics such as blood pressure and body weight. Incorporating validated indices such as the A Body Shape Index (ABSI) or waist-to-height ratio, may enhance measurement reliability (^16^).

Participants without MetS risk factors demonstrated higher adherence to smoking cessation, alcohol abstinence, regular physical activity, and low-salt diet practices. These observations suggest that consistent engagement in healthy behaviors may delay the onset of MetS risk factors among individuals with awareness of the condition. Considering the trend of earlier MetS onset, particularly among individuals in their 30s and 40s (^17^), and increasing life expectancy, long-term and continuous education regarding MetS risk and complications is essential. Direct patient-provider interactions have been shown to enhance motivation for behavior change (^18^), and personalized intervention delivered by trained healthcare professionals may be particularly effective in promoting sustained lifestyle modification (^19^).

Among participants with MetS risk factors, regular hospital visits were the most commonly reported lifestyle practice, whereas lack of knowledge regarding appropriate lifestyle behaviors was the primary barrier. This suggests a predominant reliance on pharmacological management rather than comprehensive lifestyle modification. Effective prevention of CVD complications associated with MetS requires integrated interventions encompassing both dietary modifications and regular physical activity (^13^). Therefore, practical, targeted strategies are warranted to enhance adoption and maintenance of lifestyle changes.

Although low-to-moderate intensity exercise provided some health benefits (^20^), current guidelines, including the Physical Activity Guidelines for Americans, recommend moderate-to-vigorous physical activity (MVPA) for optimal cardiometabolic outcomes (^21^). Evidence indicates that increased MVPA is more strongly associated with reduced MetS prevalence in men compared to women (^22^). Structured programs modeled on cardiac rehabilitation may facilitate adherence by combining clinical oversight with individualized exercise plans, and the long-term sustainability of such interventions should be systematically evaluated.

While sodium restriction is commonly recommended, emerging evidence suggests that both excessive and insufficient sodium intake may contribute to metabolic dysfunction, including insulin resistance and obesity (^23^). Structured dietary interventions, such as the Dietary Approaches to Stop Hypertension (DASH) diet (^24^), Mediterranean diet, and Nordic diet (^25^), which emphasize nutrient balance rather than strict sodium reduction, may provide broader metabolic benefits. Additionally, accumulating evidence highlights the role of sleep quality in metabolic health (^26^), suggesting that sleep management should be integrated into comprehensive lifestyle programs, potentially through home-based or technology-assisted interventions to enhance, especially among younger populations.

This study identified female sex, older age, and greater awareness of MetS complications as significant predictors of engagement in healthy lifestyle behaviors. MetS prevalence increases with age in both sexes (^27^), with a pronounced rise in postmenopausal women (^28^). While some studies indicated that older women with lower healthy lifestyle adherence are at elevated risk of MetS, others evidence suggests that older adults may adopt protective health behaviors more consistently (^27^). Cohort studies have further demonstrated that lifestyle interventions may yield greater reduction in MetS risk among older men compared to younger adults or women, potentially reflecting pre-existing behavioral differences (^29^). These findings underscore the importance of tailoring interventions according to both age and sex.

Participants with higher awareness of the relationship between MetS and CVD complications were more likely to engage in preventive lifestyle behaviors. Furthermore, a family history of CVD was associated with a higher prevalence of MetS risk factors. These findings hightlight the need for tailored educational interventions that provide practical, real-world examples demonstrating that effective management of MetS can reduce CVD complications, lower healthcare costs, alleviate familial burden, and improved quality of life for patients and their families. Implementing a systematic management framework that includes assessments such as microalbuminuria testing and fundus examinations within national health screening programs may facilitate early detection of CVD complications associated with MetS. Consistent monitoring beginning in the third decade of life, with follow-up during routine health checkups, would promote sustained preventive management.

Despite the strengths of this study, including a large, nationally representative sample, several limitations should be acknowledged.

First, lifestyle practices were assessed via self-report, which may introduce reporting bias and limit the objectivity of behavioral measures. Second, adults age ≥70 years were excluded due to the online survey format, potentially limiting generalizability to older populations. Third, psychological variables such as stress, known to influence MetS risk and lifestyle adherence (^30^), were not assessed. Future research should incorporate stress and sleep quality, and other behavioral determinants to inform comprehensive lifestyle intervention programs. Finally, the observed variation in lifestyle adherence by age and sex emphasizes the necessity for personalized interventions. Developing and evaluating the effectiveness of tailored programs will be critical for reducing the burden of MetS and improving long-term health outcomes.

In conclusion, this study evaluated the prevalence of MetS risk factors and adherence to healthy lifestyle behaviors among South Korean adults. Among the assessed risk factors, abdominal (central) obesity was the most prevalent within the META group; however, awareness and routine monitoring―particularly waist circumference assessment―remained limited. Given its critical role in MetS pathogenesis and CVD risk, waist circumference measurement should be emphasized through targeted public education and systematic integration into routine clinical practice.

Female sex, older age, and greater awareness of MetS-related complications were significantly associated with higher adherence to healthy lifestyle behaviors. These findings underscore the importance of tailored, demographic-specific, and preventive interventions aimed at delaying or preventing the onset of MetS and its associated CVD complications. Implementation of such strategies has the potential to reduce healthcare costs, alleviate disease burden, and improved long-term health outcomes at both individual and population levels.

## Figures and Tables

**Table:** 

①	less than 1 million won	②	less than 1 to 2 million won
③	less than 2 to 3 million won	➃	less than 3 to 4 million won
⑤	less than 4 to 5 million won	⑥	5 million won or more

**Table:** 

Hypertension (HTN)	Yes	No
Dyslipidemia (Hyperlipidemia)	Yes	No
DM	Yes	No
Drinking	Yes	No
Smoking	Yes	No
Abdomen obesity	Yes	No

**Table:** 

①	Self-employment (store, shops, restaurants, etc.)
②	Sales (office workers who work outside the office more than inside the office, salespeople, etc.)
③	Service/Customer service (clerks, bank tellers, etc.)
➃	Technical/Operational (production workers, etc.)
⑤	General office work (office workers who work inside the office more than outside the office, office workers, etc.)
⑥	Administration/Management (manager, managing director, CEO, etc.)
⑦	Professional (professors, doctors, etc.)
⑧	Public official
⑨	Teacher
⑩	College/University student (including students on leave)
⑪	Graduate student (including students on leave)
⑫	Housewife (married woman without a job)
⑬	Part time/Freelancer
⑭	None
⑮	Others

## Data Availability

datasets related to this article will be avail-able upon request to the corresponding author.

## SUPPLEMENTARY MATERIAL

### 1. Demographic characteristics

**DQ1.** Please answer your age

_______ years old

**DQ2.** Please answer your gender

① Male ② Female

**DQ3.** Please answer your highest level of education

① Middle school graduate or younger ② High school ③ College and university graduate or higher

**DQ4.** Please answer your household income (KRW)

**DQ5.** If you have suffered from or are suffering from any of the following conditions diagnosed by a doctor, please select the appropriate option

**DQ6.** Please answer with your occupation

**DQ7.** Does anyone in your family have CVD (cardiovascular disease)

① Yes ③ No

**DQ8.** Please answer whether you are married or not

① Single

② Married

③ Divorce/Bereaved

④ Others

### 2. Metabolic syndrome related questionnaire

1. Do you think you know(aware) about metabolic syndrome?

1) Know very well

2) Know it well

3) Know a little bit

4) Don’t know

5) Don’t know at all

2. Do you think that people with metabolic syndrome are more likely to develop diabetes or cardiovascular disease in the future?

1) Strongly agree

2) Somewhat agree

3) Slightly agree

4) Do not agree

5) Do not agree at all

3. Have you measured your waist circumference in the last three years?

1) Frequently

2) Occasionally

3) Rarely

4) Never

4. Have you measured your weight in the last three years?

1) Frequently

2) Occasionally

3) Rarely

4) Never

5. What lifestyle changes are you making to prevent metabolic syndrome (select all that apply)

1) Measure blood pressure

2) Routine medical checkup

3) Adequate sleep

4) Low salt diet

5) Physical activity

6) Taking vitamins

7) Taking health supplements (red ginseng, etc.)

6. What do you think are the barriers to implementing a healthy lifestyle to prevent metabolic syndrome

1) Economic burden (money or insurance)

2) Maintaining a healthy lifestyle

3) Lack of time

4) Too much to do

5) Not sure what to do

6) Lifestyle modification don’t help (changing lifestyle will reduce my risk of developing the disease)

7) Others
